# Peripheral neurolymphomatosis with tracheal asphyxia: a case report and literature review

**DOI:** 10.1186/s12883-015-0405-2

**Published:** 2015-08-23

**Authors:** Zuofeng Liu, Tao Jiang, Ni Hou, Yongqian Jia

**Affiliations:** Department of Hematology and Research Laboratory of Hematology, West China Hospital, Sichuan University, Chengdu, 610041 China; Department of Hematology, Sichuan Academy of Medical Sciences & Sichuan Provincial People’s Hospital, Chengdu, 610072 China; Department of Nuclear Medicine, West China Hospital, Sichuan University, Chengdu, 610041 China

**Keywords:** Neurolymphomatosis, Brachial plexus, Vagus nerve, Tracheal asphyxia

## Abstract

**Background:**

Neurolymphomatosis (NL) is an extremely rare disease and tracheal asphyxia due to NL has not been previously reported.

**Case Presentation:**

A 54-year-old Chinese woman with a history of diffuse large B-cell lymphoma in her first complete remission developed peripheral neuropathy and tracheal asphyxia. Neurolymphomatosis involving the right brachial plexus and the right vagus nerve was demonstrated by PET/CT, but not by MRI. She underwent urgent tracheotomy and impact chemotherapy using rituximab combined with high dose methotrexate and involved field radiotherapy. She achieved a second complete remission.

**Conclusion:**

PET/CT plays valuable role in differentiating NL from other neuropathies in patients with lymphoma. Complete remission can be achieved in NL due to large B-cell lymphoma.

## Background

Neurolymphomatosis (NL) is an extremely rare disease that can be caused by T cell, B cell or NK cell lymphoma, but most commonly B cell lymphoma. It is characterized by lymphoma cells directly infiltrating cranial nerve, peripheral nerve, nerve root and nerve plexus. NL may occur primary in the peripheral nervous system or occur as a part of systemic lymphoma [1–3]. NL may involve different parts of the peripheral nervous system, but simultaneous involvement of brachial plexus nerve and vagus nerve has not been reported in the literature. Here we report one such a case of NL simultaneously involving brachial plexus nerve and vagus that caused a medical emergency of vocal cord paralysis and trachea asphyxia.

## Case presentation

- A 54 years old female first presented to hematology department of West China Hospital in November 2012 with numbness and pain of right upper extremity lasting for 2 months. She denied any past medical history. A 5 × 5 cm mass in upper outer quadrant of right breast was palpated during the physical examination. A neurological examination showed diminished flexion and extension of right fingers. Electromyogram (EMG) showed peripheral neurogenic damage of right upper extremity. Fine needle aspiration of the mass revealed some atypical cells scattered or focally infiltrated in the breast stroma. Immunohistochemistry of the atypical cells showed diffuse large B cell lymphoma (DLBCL). CT scan of chest and bone marrow studies were negative. Cerebrospinal fluid (CSF) analysis showed no nucleated cells, protein 0.56 g/L, normal chlorides and glucose. A flow cytometry examination showed no abnormal cells. She received 2 courses R-hyperCVAD(rituximab + scheme A: cyclophosphamide + vindesine + doxorubicin + dexamethasone/scheme B: methotrexate + cytarabine), 2 courses R-CHOP (rituximab + cyclophosphamide + vindesine + liposomal doxorubicin + prednisone) and 4 times intrathecal preventive chemotherapy (methotrexate + cytarabine + dexamethasone). Protein level of CSF fluctuated between 0.54-0.61 g/L and no abnormal cell was detected by flow cytometry. She was considered in complete remission. The patient was stable during regular follow-ups.

**Fig. 1:**
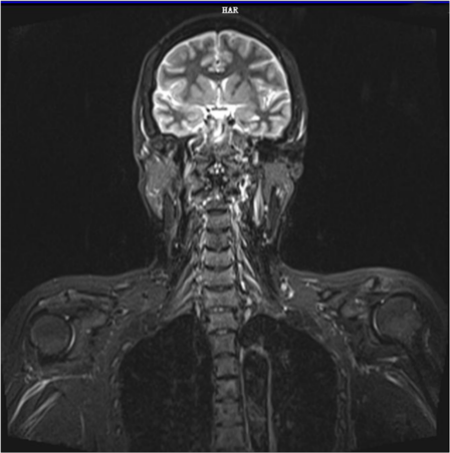
MRIs of nerve plexus showing no apparent changes

**Fig. 2:**
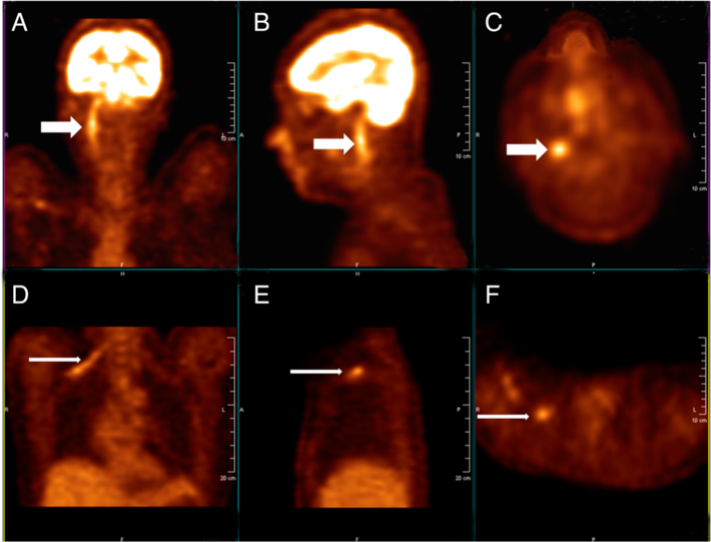
PET/CT images before treatment showing involvement of right vagus nerve and brachial plexus (detailed images). **a, b, c** show abnormally high glucose metabolism area of the right vagus nerve running zone; **d, e, f** show abnormally high glucose metabolism area of right brachial plexus running zone; **a**, **d** are coronal sections; **b**, **e** are sagittal sections; **c**,**f** are cross sections; white arrows direct at lesions in nerves

**Fig. 3:**
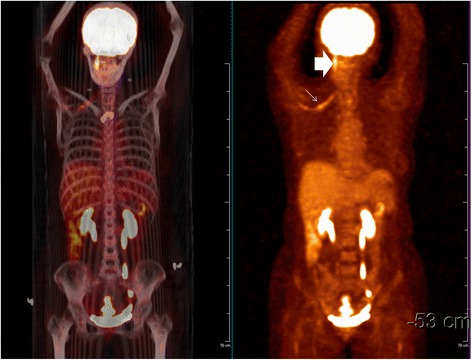
PET/CT before treatment showing involvement of right vagus nerve and brachial plexus (whole body images). Wide white arrow indicates high glucose metabolism area of the right vagus nerve running zone; fine white arrow indicates high glucose metabolism area of the right brachial plexus running zone

In February 2014, right upper extremity numbness reoccurred and developed nonproductive cough, and shortness of breath which worsened at night. She was hospitalized. Tactile hypoesthesia and hypoalgesia of right forearm and dorsal ulnar side of right palm were also noted again. Her lungs were clear by auscultation and a chest CT scan was normal. She was given an empirical treatment using antibiotics and glucocorticoid. Her symptoms deteriorated. She developed marked numbness and severe pain in the right arm, also strength reduction and limited activities. She showed paroxysmal choke to cough and hoarse voice. Twenty days after admission she developed acute respiratory distress and stridor. An urgent laryngoscopy revealed bilateral vocal cord paralysis and laryngeal obstruction. An emergency tracheotomy was performed due to the trachea asphyxia. Enhanced MRIs of head neck and thorax were unremarkable (Fig. 1). PET/CT, however, showed significant abnormally in the area of the right vagus nerve running zone and right brachial plexus running zone, indicating tumor invasion (Figs. 2 and 3). CSF protein level was 0.52 g/L and no abnormal cell was detected by flow cytometry. Considering the patient’s medical history of lymphoma, symptoms and signs as well as the PET/CT results, a diagnosis of peripheral neurolymphomatosis (involving the right brachial plexus nerve, vagus nerve) and extranodal relapse of DLBCL was established after other factors, such as chemotherapy, infections, tumor compression and paraneoplastic neuropathy had been taken into account.

**Fig. 4:**
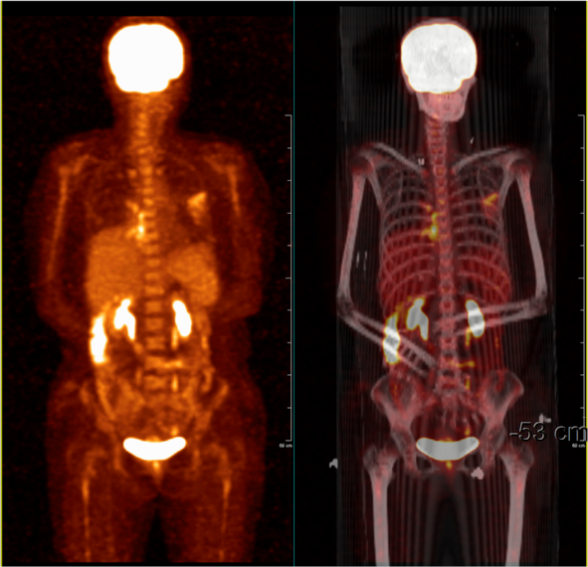
Whole body PET/CT after treatment showing resolution of high metabolic foci

The patient was given five courses R-DHAP(rituximab + cisplatin + cytarabine + dexamethasone) and high dose methotrexate impact chemotherapy. The patient received 30-35Gy involved field radiotherapy. All her symptoms resolved and her tracheostomy tube was removed. A follow-up PET/CT showed complete remission of the tumor involvement in the right vagus and right brachial plexus nerve (Fig. 4). She remains stable as of now.

## Discussion

Neurolymphomatosis (NL) is a rare condition with lymphoma infiltrating parts of the peripheral nerve system including cranial nerves. Only a few cases have been reported and the exact incidence is unclear. It was estimated that NL may occur in 0.2 % of all non-Hodgkin’s lymphoma (NHL) [1]. NHL is responsible for over 90 % of NL and NL rarely occurs in Hodgkin’s lymphoma or lymphoid leukemia. It has diverse clinical manifestations depending on the location of involvement. Delayed diagnosis or misdiagnosis is common and there is significant variations in treatment response and prognosis [1–4].

NL may affect the peripheral nerves, nerve roots and cranial nerves. Multiple nerve involvement is more common than single nerve involvement. Lumbosacral plexus nerve, horsetail nerve and sciatic nerve are common sites of involvement [5–7]. In this case the NL occurred during her large B-cell lymphoma relapse and manifested as brachial plexus and vagus nerve. The tumor infiltration in the peripheral nervous system caused life-threatening bilateral vocal cord paralysis and acute upper and real obstruction, which resolved after chemo and radiation therapy of her underlying large B-cell lymphoma. To our knowledge, such an acute airway obstruction as a complication of NL has not been previously reported in the literature.

The differential diagnosis of neurological symptoms in patients with lymphoma includes inflammatory neuropathy, drug-induced neuropathy, tumor compression and paraneoplastic syndrome [8]. It is also important to include NL in the differential diagnosis. Electromyography is sensitive to detecting peripheral neuropathy, but it is not specific for NL. Direct nerve biopsy is considered the the gold standard for the diagnosis [7]. However, it is invasive and false negative results occur due to sampling errors. Ramirez-Zamora [9] reported 1 case of NL with negative nerve biopsy that was later confirmed by autopsy. NL is easily misdiagnosed or missed due to its rarity and complex clinical manifestations. An earlier retrospective study showed that about half of the cases were only diagnosed by autopsy [1]. Casselberry [10] reported one 64-year-old NL patient presented with aggravating neurological dysfunction that had been misdiagnosed as Guillain-Barré syndrome and inflammatory demyelination. The patient died after 3 months of ineffective treatment and was later found to have NL by autopsy.

About 60 % of patients with NL per reported to have elevated protein level of CSF [2]. Repeated CSF examinations of our case found slightly elevated protein as well. However this is nonspecific and is sensitive. Enhanced MRI is a very useful tool in neurology practice. However it appears to have limited role in detecting NL peripheral nerve involvement, as demonstrated in our case report. PET/CT is far more superior than MRI. It is generally believed that MRI has lower sensitivity than PET/CT depending on the thickening of involved nerve, nerve root/bundle with or without contrast enhancement [1, 2]. PET/CT has higher sensitivity and specificity for NL and the abnormally high glucose metabolism can be used to follow therapeutic response [11–13]. We feel that a PET/CT should be performed in all patients suspected of NL. If a convincingly positive test result is obtained, the treatment for the underlying lymphoma should be instituted immediately without a nerve biopsy.

The prognosis of NL varies greatly among different patients. It’s reported that the median survival time was ten months and 3 year overall survival rate was 24 % [2]. One case was reported to have survived over 31 years [14]. Currently high dose methotrexate plus involved field radiotherapy is the treatment of choice for NL [1–3, 7]. Rituximab, hematopoietic stem cell transplantation and plasma exchange have also been tried to treat NL with variable success [3, 11, 15]. We used 5 cycles of rituximab combined with high dose methotrexate impact chemotherapy for this patient who achieved complete clinical remission based on her symptoms and follow-up PET/CT studies. However her long-term survival needs further observation.

## Conclusion

NL is a rare complication of lymphoma with highly variable clinical manifestations depending on the locations of peripheral nerve involvement. Early diagnosis can be challenging. EMG and CSF protein level may be helpful but nonspecific. Enhanced MRI is of limited value. PET/CT appears to be sensitive and specific for NL diagnosis. Complete remission of lymphoma can still be achieved despite the development of NL.

### Consent

Written informed consent was obtained from the patient for publication of this Case report and any accompanying images. A copy of the written consent is available for review by the Editor of this journal.
